# Colon and liver tissue damage detection using methylated SESN3 and PTK2B genes in circulating cell-free DNA in patients with acute graft-versus-host disease

**DOI:** 10.1038/s41409-020-01090-z

**Published:** 2020-10-20

**Authors:** Miguel Waterhouse, Sandra Pennisi, Dietmar Pfeifer, Max Deuter, Nikolas von Bubnoff, Florian Scherer, Tim Strüssmann, Claudia Wehr, Justus Duyster, Hartmut Bertz, Jürgen Finke, Jesus Duque-Afonso

**Affiliations:** 1 Department of Hematology, Oncology and Stem Cell Transplantation, Freiburg University Medical Center, Freiburg, Germany; 2 Department of Hematology and Oncology, Medical Center, University of Schleswig Holstein, Lübeck, Germany

**Keywords:** Acute myeloid leukaemia, Translational research

## Abstract

Cell-free DNA (cfDNA) has been investigated in acute graft-versus-host disease (aGvHD) following allogeneic cell transplantation (HSCT). Identifying the tissue of origin of cfDNA in patients with aGvHD is relevant particularly when a biopsy is not feasible. We investigate the cfDNA tissue of origin in patients with aGvHD using methylated gene biomarkers. Patients with liver, colon, or skin aGvHD (*n* = 28) were analyzed. Liver- and colon-derived cfDNA was measured using a colon- (SESN3) and liver (PTK2B)-specific methylation marker with digital droplet PCR. A statistically significant difference (*p* < 0.001) in PTK2B and SESN3 concentration was observed between patients with colon or liver GvHD and the control group. For SESN3 and PTK2B the area under the curve in the receiver-operating characteristic (ROC) space was 0.952 (95% CI, 0.888–1 *p* < 0.001) and 0.971 (95% CI, 0.964–1 *p* < 0.001), respectively. Thresholds to differentiate aGvHD from non-aGvHD in colon were 0 (sensitivity: 0.905; specificity: 0.989) and liver 1.5 (sensitivity: 0.928; specificity: 0.910). Clinical improvement of liver or colon aGvHD resulted in PTK2B and SESN3 reduced concentration. Whereas, in those patients without improvement the PTK2B and SESN3 level remained stable or increased. The PTK2B liver-specific marker and the SESN3 colon-specific marker and their longitudinal analysis might improve aGvHD detection.

## Introduction

Circulating cell-free DNA (cfDNA) isolated from plasma or serum has been used for different diagnostics applications such as mutation detection in solid tumors [1], fetal chromosomal aberrations [2], and organ rejection screening in solid organ transplantation among others [3]. cfDNA can be easily obtained without the need of invasive procedures resulting in a valuable source of genetic material. However, cfDNA is a complex mixture and several organs might contribute to cfDNA constitution [4], therefore establishing the tissue of origin is clinically relevant albeit challenging to perform. Furthermore, cfDNA derived from different tissues might have a different dynamic that in turn may be influenced by physiological or pathological conditions [5]. Placentally derived cfDNA has been proved useful for detection of fetal trisomy 18 [6]. In addition, cfDNA derived from erythroid precursors can be used to characterize different types of anemia and their response to treatment [7].

Methylation deconvolution and genome-wide bisulfite sequencing represent a method to determine the tissue of origin of cfDNA [8]. However, this approach is expensive and requires long turn-around times. Alternative approaches based on methylation-specific PCR for the detection of highly methylated organ-specific genes have been proposed [9].

In our previous study, we were able to show the relation between cfDNA and allogeneic cell transplantation (HSCT)-related complications such as acute graft-versus-host disease (aGvHD) and relapse [10]. Patients with aGvHD showed a higher percentage of recipient cfDNA when compared to controls. In addition, in those patients with aGvHD clinical improvement a decrease in the recipient cfDNA percentage was observed, while in patients without aGvHD clinical improvement, the recipient cfDNA percentage remained stable or even increased. However, it was not possible to establish the tissue of origin of cfDNA, reducing in this way their potential clinical usefulness. Identifying the tissue of origin of cfDNA in patients with a clinical diagnosis of liver or colon aGvHD is relevant because diagnosis of aGvHD by histology, considered the gold standard, requires invasive tissue sampling that is often precluded by infection risk, thrombocytopenia, and other post-transplant complications [11]. In the present report, we aim to investigate the cfDNA tissue of origin in patients that develop aGvHD after HSCT using methylated gene biomarkers. In addition, we analyzed the recipient organ-specific cfDNA kinetics after aGvHD treatment. To our knowledge, this is the first report to describe the utility of detecting gene methylation in cfDNA as biomarkers in an HSCT setting.

## Patients and methods

### Patient characteristics

A total of 73 patients undergoing HSCT from the University of Freiburg Medical Center were analyzed. The study group diagnosed with either acute or late onset aGvHD consisted of 28 patients. Within this group, 13 patients had follow-up samples with a mean of three samples per patient. Healthy donors (*n* = 10) and transplanted patients (*n* = 11) without transplant-related complications including aGvHD were used as a control group. Patients with chemotherapy-induced liver damage (*n* = 5) and patients with diarrheal disorders after allogeneic or autologous stem cell transplantation including neutropenic colitis, drug-induced and *Clostridium difficile* infection (*n* = 9) were also included as controls. Patients with colorectal cancer (CRC) and liver metastases (*n* = 7) were used as controls for patients with known colon and liver tissue damage. The transplantation procedure and aGvHD prophylaxis were performed as previously described [12]. Diagnosis of aGvHD was defined based on standard clinical criteria and by histology when possible. The aGvHD onset day was defined as the time of starting immunosuppressive therapy. The clinical characteristics of the patients with aGvHD included in the study are listed in Table 1. Two 10-ml peripheral blood aliquots were collected in EDTA tubes. Peripheral blood processing, plasma isolation, and storage were performed as previously described [10].

**Table 1:** Patients with aGvHD. Clinical characteristics.

Gender male/female	15/13
Mean age at transplant (range)	53 (20–75)
Donor type	
Related	11
Non related	17
Conditioning regimen	
Myeloablative	8
Reduced toxicity	20
Initial diagnosis	
Acute myeloid leukemia	18
Acute lymphoblastic leukemia	2
Non-Hodgkin lymphoma	2
Multiple myeloma	2
Others	4
GvHD target organ^a^	
Skin	4
Liver	10
Colon^b^	14
Mean sampling time in days^c^ (range)	15 (0–55)
Acute GvHD grade	
Grade I–II	11
Grade III–IV	17
Acute GvHD treatment response	
Steroid-sensitive	8
Steroid-refractory	20
GvHD prophylaxis	
CyA/MMF/ATG	15
CyA/MMF	8
CyA/MTX	2
Everolimus/MMF/ATG	1
CyA/MMF/cyclophosphamide	1
CyA/Alemtuzumab	1

*CyA* cyclosporin A, *MMF* mycophenolate mofetil, *ATG* anti-thymocyte globulin.

^a^aGvHD was histologically confirmed in all patients with colon GvHD. In patients with liver or skin aGvHD the diagnosis was clinically done.

^b^In all patients, aGvHD was located in the lower gastrointestinal tract.

^c^Time between inmmunosuppression start and blood sampling.

### DNA isolation and chimerism testing

DNA was extracted from peripheral blood and plasma samples using the Qiasymphony miniDNA kit according to the manufacturer’s instructions (Qiagen GmbH, Hilden, Germany).

Chimerism status in blood and plasma was performed simultaneously in the same sample and analyzed using the QX200 Droplet Digital PCR system (Bio-Rad Laboratories, Munich, Germany). The panel of insertion/deletion polymorphic markers used for chimerism testing and their interpretation has been previously described [13].

### DNA bisulfite conversion and droplet digital PCR for liver and colon markers

The DNA extracted from plasma or peripheral blood was subjected to bisulfite conversion and subsequent purification using the EZ DNA Methylation-Lightning kit (Zymo Research, Freiburg, Germany) according to the kit protocol. The droplet digital PCR assays for liver-specific (PTK2B) and colon-specific (SESN3) methylation markers were performed as previously described by Gai et al. with slight modifications [9]. Briefly, the dPCR reaction volume was 20 µL composed of 10 µL of dPCR supermix for probes (Bio-Rad Laboratories, Munich, Germany), 6 µL of bisulfite-converted DNA and primer and probes concentration as already described. Each reaction mixture was partioned into ~20,000 droplets using a droplet generator (Bio-Rad Laboratories, Munich, Germany) and then cycled under the following conditions: 95 °C for 10 min, followed by 40 cycles of 94 °C for 30 s, 60 °C or 56 °C for the liver or colon assay respectively, for 1 min and one final cycle of 98 °C for 10 min. Cycled droplets were read in a QX200 droplet-reader and the analysis of the dPCR data was performed using the QuantaSoft analysis software (Bio-Rad Laboratories, Munich, Germany). The threshold between the positive and negative droplet clusters was manually set for each fluorochrome channel. Absolute number of the target bisulfite-converted DNA copies per ml plasma was calculated according to the formula described by van Ginkel et al. [14]. Patients with CRC and liver metastasis have been described to have increased colon- and liver-derived cfDNA when compared to controls [15]. Therefore, samples from patients with active CRC and liver metastasis were included to optimize DNA extraction from plasma, bisulfite conversion, and PCR conditions.

### Statistical analysis

Common statistical parameters were calculated using Analyse-it software version 5.51 (Analyse-it, Leeds, United Kingdom). Correlation coefficients were calculated by the Spearman rank correlation coefficient analysis. For the comparison of qualitative or quantitative variables without a normal distribution, the Mann–Whitney, Wilcoxon signed-rank test, or Kruskal–Wallis tests were used. Quantitative variables were analyzed with the Student paired *t*-test or Fisher’s exact test in the case of small numbers. All tests were two-sided, accepting *p* ≤ 0.05 as indicating a statistically significant difference. General performance of the test was analyzed by plotting the true-positive rate (sensitivity) and the false-positive rate (1-specificity) in a receiver-operating characteristic (ROC) space. Youden index was used to estimate the optimal threshold value for each assay.

## Results

### Chimerism status of circulating cfDNA in patients with aGvHD

**Fig. 1:**
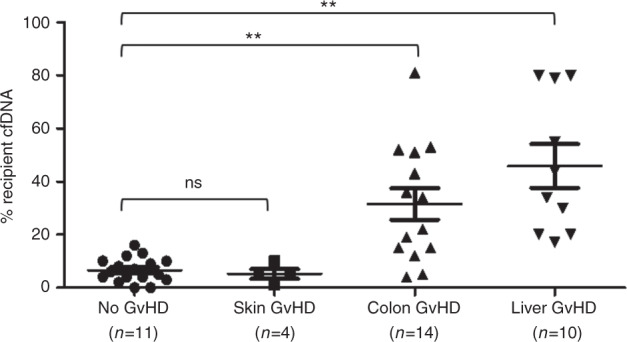
Percentage of recipient cfDNA in patients with skin (■), liver (▼), or colon (▲) aGvHD. There was a significant difference in the mean percentage of recipient cfDNA between patients with either liver or colon aGvHD compared with patients without aGvHD (**). No significant difference was observed between patients with skin aGvHD and patients without HSCT complications (ns).

After transplantation all patients with aGvHD (*n* = 28) showed a mixture of donor and recipient DNA in the isolated cfDNA. The mean percentage of recipient-derived cfDNA in the control group, namely patients (*n* = 11) without transplant-related complications, was 6.8% (95% confidence interval (CI): 4.3–9%). When patients were grouped according to the aGvHD target organ (skin, liver, or colon), a significant difference in plasma chimerism was observed between patients with liver (mean recipient-derived cfDNA: 45.9%; 95% CI: 27.3–65.4%, *p* < 0.001) and colon aGvHD (mean recipient-derived cfDNA: 31.6%; 95% CI: 18.7–44.5%, *p* < 0.001) and the control group. No significant difference in the plasma chimerism was detected in patients with skin aGvHD (mean recipient-derived cfDNA: 5.3%; 95% CI: 1–11.1%, *p* = 0.467) and the control group. When the true-positive fraction (sensitivity) and the false-positive fraction (1-specificity) were plotted in a ROC space, the area under the curve (AUC) was 0.886 (95% CI; 0.756–0.97 *p* < 0.001) and 0.911 (95% CI; 0.791–1.02 *p* < 0.001) for those patients with colon or liver aGvHD, respectively. The optimal threshold to discriminate liver or colon aGvHD from non-aGvHD was 10% of recipient-derived cfDNA (sensitivity: 0.857; specificity: 0.844) (Supplementary Fig. 1). All patients with aGvHD and mixed chimerism in cfDNA were complete donor in peripheral blood mononuclear cells (data not shown). The percentage of recipient-derived cfDNA according to the aGvHD target organ is depicted in Fig. 1.

### Liver- and colon-derived cfDNA after HSCT

The main contributor of DNA isolated from plasma of peripheral blood are disrupted white blood cells. Therefore, to estimate the potential contribution to the cfDNA of DNA derived from leukocytes, we performed the liver- (PTK2B) and colon-specific (SESN3) assays in bisulfite-converted DNA from several ficoll-isolated leukocytes of transplanted patients with normal leukocyte counts. No positive droplets were detected in the liver- and colon-specific assays when using the bisulfite-converted DNA isolated from these samples (data not shown).

The mean concentration of PTK2B in patients with liver aGvHD was 2.79 (log PTK2B copies/ml plasma; 95% CI: 2.57–3.02) within the healthy donor group, which was 0.61 (log PTK2B copies/ml plasma; 95% CI: 0.13–1.09). Regarding the liver-derived cfDNA, a statistically significant difference was found between patients with either liver aGvHD, CRC with liver metastasis (mean PTK2B concentration 2.25 log PTK2B copies/ml plasma; 95% CI: 1.87–2.66), or drug-induced liver damage (mean PTK2B concentration 2.03 log PTK2B copies/ml plasma; 95% CI: 1.04–2.7) when compared to healthy donors (*p* < 0.001). Meanwhile, in patients with colon aGvHD the mean SESN3 concentration was 1.45 (log SESN3 copies/ml plasma; 95% CI: 1.21–1.69) while in the healthy donor control group no SESN3 positive droplets were detected. Again, a statistically significant difference was found in the SESN3 concentration in patients with either colon aGvHD, CRC (mean SESN3 concentration 1.41 log SESN3 copies/ml plasma; 95% CI: 0.99–1.82) or diarrhea after transplantation not related to colon aGvHD (mean SESN3 concentration 0.45 log SESN3 copies/ml plasma; 95% CI: 0.01–0.9) when compared with healthy controls (*p* < 0.001). For the liver- and colon-specific marker, no significant difference was found between healthy controls and patients without liver or colon aGvHD (*p* = 0.362 and *p* = 1, respectively).

**Fig. 2:**
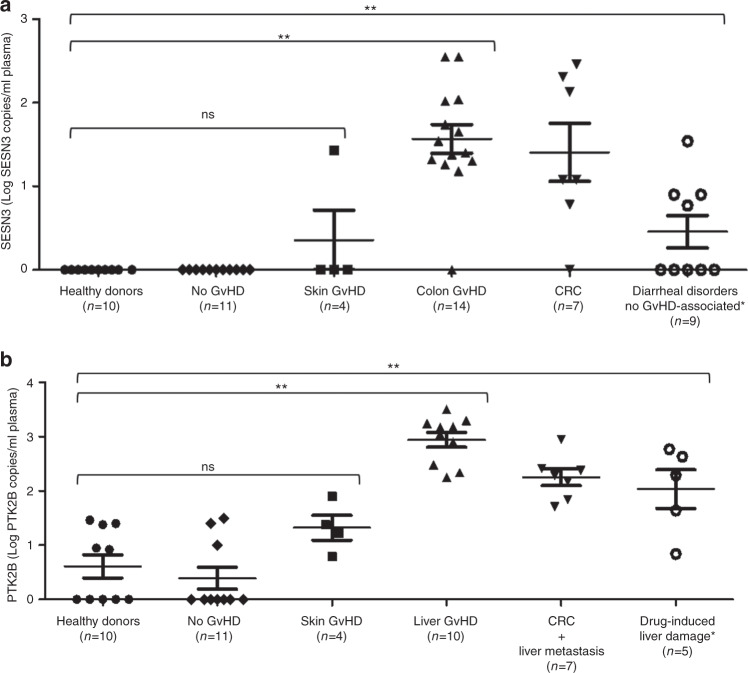
Colon- and liver-derived cfDNA assessed on organ-specific methylated genes by dPCR. **A** SESN3 cfDNA levels in patients with aGvHD (colon (▲) and skin (■), patients with CRC (▼) and patients with diarrheal disorders not associated with GvHD (○) (*neutropenic colitis, drug-induced and *Clostridium difficile* infection). A significant difference (**) in the SESN3 levels was observed between patients with either colon aGvHD, CRC, or diarrhea not associated with GvHD when compared to healthy donors (●) or patients without GvHD (♦). There was no significant difference (ns) between patients with skin aGvHD and healthy controls. **B** PTK2B cfDNA levels in patients with aGvHD (liver (▲) and skin (■)), patients with CRC and liver metastases (▼), and patients with drug-induced liver damage (○) (*conditioning regimen and high-dose chemotherapy before autologous transplantation). A significant difference (**) in the PTK2B levels was observed between patients with either liver aGvHD, liver metastases, or drug-induced liver damage when compared to healthy donors (●) or patients without GvHD (♦). There was no significant difference (ns) between patients with skin aGvHD and healthy controls.

In a small group of four patients with only skin aGvHD the mean value of SESN3 and PTK2B was 0.353 and 1.323, respectively (log SESN3/PTK2B copies/ml plasma). No significant difference was observed between patients with skin aGvHD and healthy controls. Within this last group of patients with skin aGvHD, one patient subsequently developed colon and liver aGvHD. Results for PTK2B and SESN3 in patients with colon, skin, and liver aGvHD are depicted in Fig. 2.

A moderate correlation was observed between the PTK2B concentration and the percentage of recipient cfDNA in patients with liver aGvHD (Spearman *s* = 0.492), while no correlation between SESN3 and plasma mixed chimerism in patients with colon aGvHD was detected (Spearman *s* = 0.115) (Supplementary Fig. 2).

To analyse the general performance of the liver and colon assays, ROC curves were plotted using samples from transplanted patients without evidence of aGvHD. For the colon- and liver-specific markers, the AUC in the ROC curves was 0.952 (95% CI, 0.888–1 *p* < 0.001) and 0.971 (95% CI, 0.964–1 *p* < 0.001), respectively. ROC curves are depicted in Supplementary Fig. 3. Nevertheless, patients undergoing HSCT may develop subclinical aGvHD. Therefore, we repeat the ROC analysis using a healthy control group. When plotting the ROC space using healthy donors, the colon- and liver-specific markers, the AUC in the ROC curves was 0.961 (95% CI, 0.891–1 *p* < 0.001) and 0.980 (95% CI, 0.977–1 *p* < 0.001), respectively.

**Fig. 3:**
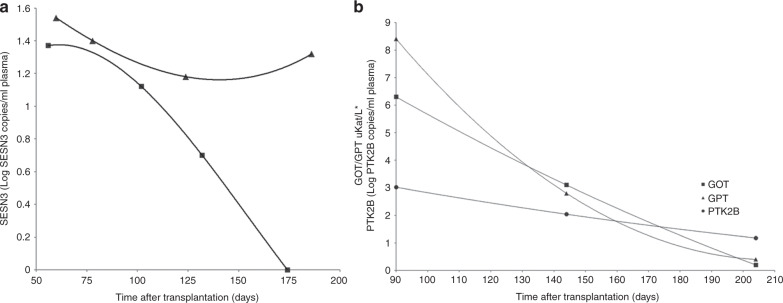
Representative example of SESN3 and PTK2B kinetics. **A** SESN3 in two patients with colon aGvHD, with clinical improvement after treatment (■) and a patient refractory to treatment (▲). **B** PTK2B (●) kinetic in a patient with successful liver aGvHD treatment and liver enzymes normalization (■ GOT, ▲GPT). *Liver enzymes (GOT, GPT) are expressed in uKat/L to fit the same PTK2B unit range.

The optimal thresholds to differentiate aGvHD from non-aGvHD in colon were 0 (log SESN3 copies/ml plasma; sensitivity: 0.905; specificity: 0.989) and liver 1.5 (log PTK2 copies/ml plasma; sensitivity: 0.928; specificity: 0.910), respectively.

Interestingly, in 7 out of 14 patients with colon aGvHD and normal liver enzymes values, the PTK2B concentration was above the established threshold. The mean PTK2B in this last group of patients was 2.4 (log PTK2B copies/ml plasma).

### PTK2B and SESN3 concentration follow-up analysis

Follow-up samples were available for 4 and 7 patients with either liver or colon aGvHD, respectively (mean follow-up: 99.8, range: 30–270 days). In line with our previous study, in patients with aGvHD and available follow-up samples two different kinetics were observed for the colon- and liver-specific markers. In the group of patients with liver aGvHD and clinical improvement as shown by liver enzymes normalization, PTK2B concentration gradually decreased, eventually reaching levels below the established threshold. In those patients without improvement or aGvHD worsening the PTK2B level remained stable or even increased. A similar pattern was observed for the SESN3 concentration in those patients with colon aGvHD. Furthermore, in two patients that died with colon aGvHD, SESN3 concentration remained stable or increased (Fig. 3).

## Discussion

GvHD is a life-threatening transplant-related complication affecting several organs such as skin, liver, and colon [16]. To confirm the clinical diagnosis, a biopsy is often required although invasive tissue sampling is not always feasible due to thrombocytopenia and the risk of infection among other complications. Therefore, cfDNA might be an alternative valuable source for aGvHD detection. In a previous study that included 110 patients, we observed that all patients with aGvHD showed a higher proportion of recipient-derived cfDNA when compared with patients without aGvHD [10]. In addition, cfDNA kinetics were correlated with the aGvHD clinical course, suggesting that the recipient cfDNA could be derived by cell disruption in aGvHD target organs. However, it was not possible to identify the tissue of origin of the cfDNA and in turn correlate aGvHD clinical course with a tissue-specific circulating nucleic acid.

Recently, using a methylation-specific PCR-based method, Gai et al. were able to show tissue-specific DNA methylation markers of liver- and colon-derived DNA in CRC and hepatocellular carcinoma [9]. In this study, tissue-derived cfDNA specificity was demonstrated by performing the SESN3 colon-specific marker and the PTK2B liver-specific marker in several tissues such as lung, esophagus, stomach, pancreas, and heart among others. Using this approach, we were able to show a significant difference in PTK2B and SESN3 concentration in patients with liver and colon aGvHD, respectively, when compared to controls. Furthermore, successful aGvHD treatment resulted in reduced PTK2B or SESN3 concentration over time suggesting that indeed cfDNA derived either from the liver or colon, respectively. Although in a different context, the PTK2B and SESN3 concentration in patients with CRC and liver metastases were comparable to patients with liver or colon aGvHD. This observation further supports that PTK2B and SESN3 are tissue-specific markers. The high diagnostic accuracy for both markers, represented by the AUC in the ROC curve and the high sensitivity and specificity of the established thresholds found in our study suggest that PTK2B and SESN3 are suitable diagnostic adjuncts for screening patients with either liver or colon aGvHD. High sensitivity assays result in a low false-negative rate, which in turn minimizes the probability of missing patients with aGvHD. Nevertheless, prospective analysis in a larger patient cohort is needed to establish accurately the true false-negative rate. It is worth noting that in half of the patients with colon aGvHD and normal liver enzymes the PTK2B concentration was raised above the threshold value. In addition, we also observed in patients with only skin aGvHD a slight increase in SESN3 and PTK2B. Taken together, this data suggest that aGvHD might affect several organs simultaneously but is clinically detected in a discrete number of organs. Due to the reduced number of patients with aGvHD in our cohort and limited follow-up samples, our results should be interpreted with caution.

We also found only a moderate correlation between the recipient cfDNA percentage and the PTK2B concentration and no correlation with the SESN3 concentration. A reduced number of patients with recipient-derived cfDNA below 10% showed PTK2B or SESN3 values above the established threshold. Therefore, when screening patients for aGvHD detection, plasma cfDNA chimerism, PTK2B and SESN3 concentration should be performed together to increase the diagnostic yield.

Although the approach and the markers used in our study may be useful for aGvHD detection, we were able to show that other sources of tissue damage may increase the concentration of PTK2B and SESN3. Conditions such as viral reactivation [17], drug toxicity [18], tumoral organ infiltration [19], and sinusoidal obstruction syndrome [20] among others may result in elevated PTK2B and SESN3 concentrations. Additional studies using these markers are granted to investigate PTK2B and SESN3 variations in other transplant-related complications other than aGvHD as infections and chemotherapy-induced organ toxicity. In our study we also established PTK2B and SESN3 values in a healthy population with demographic characteristics similar to the study population. Nevertheless, other conditions such as age, gender, or ethnic group may influence PTK2B and SESN3 reference values. Therefore, a larger study including different healthy volunteer groups should be perform to accurately settle reference values for these markers.

One caveat of our study is that colon- or liver-aGvHD detection before onset of clinical symptoms was not feasible due to the lack of prospective collection of patient samples. A prospective study using these markers is needed to investigate the early detection rate of liver and colon aGvHD. Early aGvHD detection allows prompt clinical intervention, which in turn may result in an improved HSCT outcome [21].

In brief, the PTK2B liver-specific marker and the SESN3 colon-specific marker in combination with cfDNA chimerism and their longitudinal analysis might improve aGvHD detection, particularly when a biopsy or other invasive procedures are not possible due to transplant-related complications.

## Supplementary information

Supplementary figure legends

Supplmentary figure 1

Supplementary figure 2

Supplementary figure 3
