# Correction: Colon and liver tissue damage detection using methylated SESN3 and PTK2B genes in circulating cell-free DNA in patients with acute graft-versus-host disease

**DOI:** 10.1038/s41409-021-01405-8

**Published:** 2021-08-19

**Authors:** Miguel Waterhouse, Sandra Pennisi, Dietmar Pfeifer, Max Deuter, Nikolas von Bubnoff, Florian Scherer, Tim Strüssmann, Claudia Wehr, Justus Duyster, Hartmut Bertz, Jürgen Finke, Jesus Duque-Afonso

**Affiliations:** 1 Department of Hematology, Oncology and Stem Cell Transplantation, Freiburg University Medical Center, Freiburg, Germany; 2 Department of Hematology and Oncology, Medical Center, University of Schleswig Holstein, Lübeck, Germany

**Keywords:** Acute myeloid leukaemia, Translational research

Correction to: *Bone Marrow Transplantation* 10.1038/s41409-020-01090-z, published online 20 October 2020

The article “Colon and liver tissue damage detection using methylated SESN3 and PTK2B genes in circulating cell-free DNA in patients with acute graft-versus-host disease”, written by Miguel Waterhouse et al., was originally published Online First without Open Access. After publication in volume 56, issue 2, page 327–333 the author decided to opt for Open Choice and to make the article an Open Access publication. Therefore, the copyright of the article has been changed to © Author(s) 2021 and the article is forthwith distributed under a Creative Commons Attribution 4.0 International License, which permits use, sharing, adaptation, distribution, and reproduction in any medium or format, as long as you give appropriate credit to the original author(s) and the source, provide a link to the Creative Commons licence, and indicate if changes were made. The images or other third-party material in this article are included in the article’s Creative Commons licence, unless indicated otherwise in a credit line to the material. If material is not included in the article’s Creative Commons licence and your intended use is not permitted by statutory regulation or exceeds the permitted use, you will need to obtain permission directly from the copyright holder. To view a copy of this licence, visit http://creativecommons.org/licenses/by/4.0.Open access funding enabled and organized by Projekt DEAL.

