# Supplementary material for: Colon and liver tissue damage detection using methylated SESN3 and PTK2B genes in circulating cell-free DNA in patients with acute graft-versus-host disease
Source: Bone Marrow Transplant. 2020 Oct 20;56(2):327–33. doi: 10.1038/s41409-020-01090-z (PMC8376639; doi:10.1038/s41409-020-01090-z)
Supplement: Supplementary file 1 — Supplementary figure legends [file 41409_2020_1090_MOESM1_ESM.docx]

**Supplementary figure legends**

**Supplementary figure 1.**

Receiver-operating characteristic (ROC) of cfDNA in patients with liver (A) or colon (B) aGvHD. The diagonal line corresponds to random chance. Sensitivity and specificity curves of cfDNA at different percentages of recipient cfDNA (C). Youden index (vertical line) was calculated to establish the optimal threshold to discriminate aGvHD from non-aGvHD.

**Supplementary figure 2.**

Correlation of recipient derived cfDNA and levels of PTK2B concentration in patients with liver aGvHD (A) or levels of SESN3 concentration in patients with colon aGvHD (B).

**Supplementary figure 3.**

Receiver-operating characteristic (ROC) of PTK2B in patients with liver (A) or SESN3 in patients with colon (B) aGvHD. The diagonal line corresponds to random chance.
