# Supplementary figures and images for: Colon and liver tissue damage detection using methylated SESN3 and PTK2B genes in circulating cell-free DNA in patients with acute graft-versus-host disease
Source: Bone Marrow Transplant. 2020 Oct 20;56(2):327–33. doi: 10.1038/s41409-020-01090-z (PMC8376639; doi:10.1038/s41409-020-01090-z)

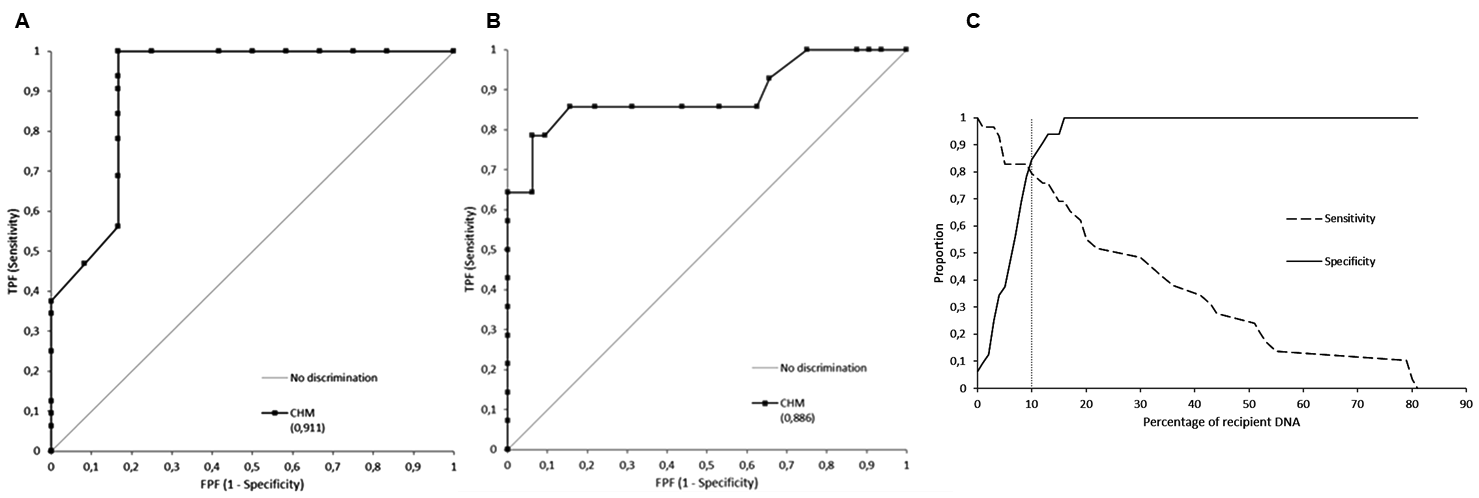

Supplement: Supplementary file 2 — Supplmentary figure 1 [file 41409_2020_1090_MOESM2_ESM.tif]

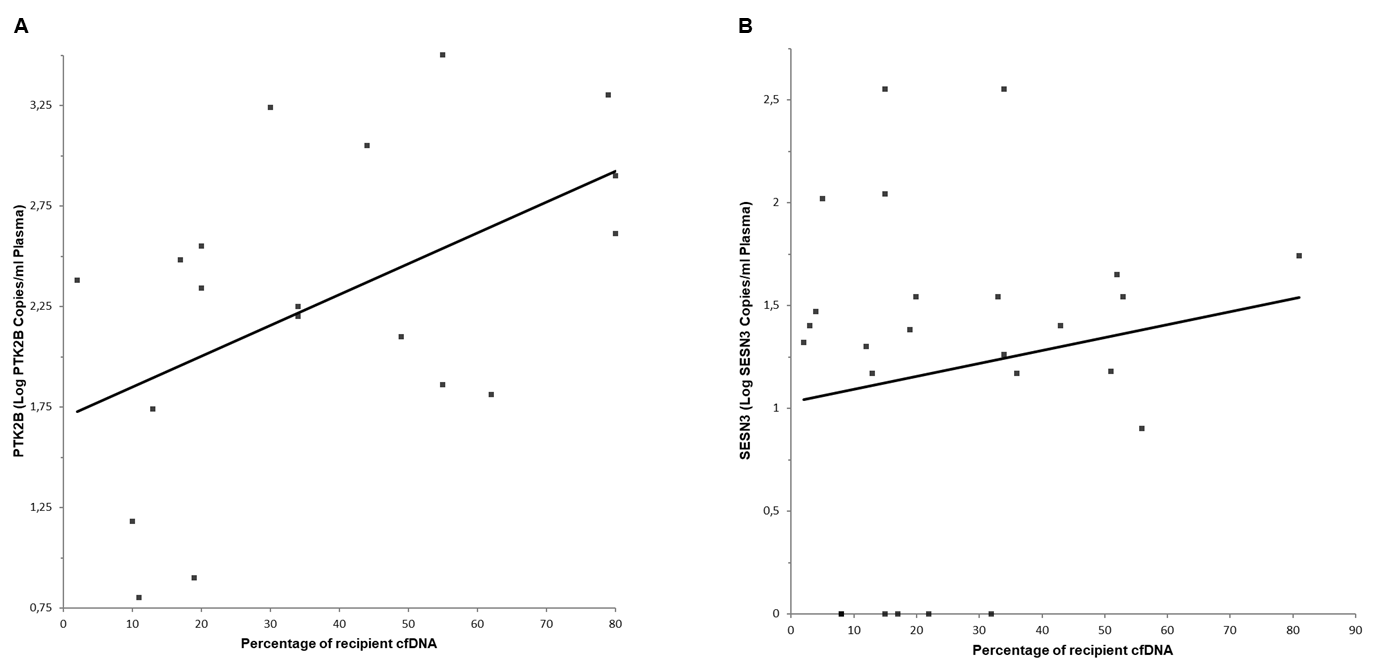

Supplement: Supplementary file 3 — Supplementary figure 2 [file 41409_2020_1090_MOESM3_ESM.tif]

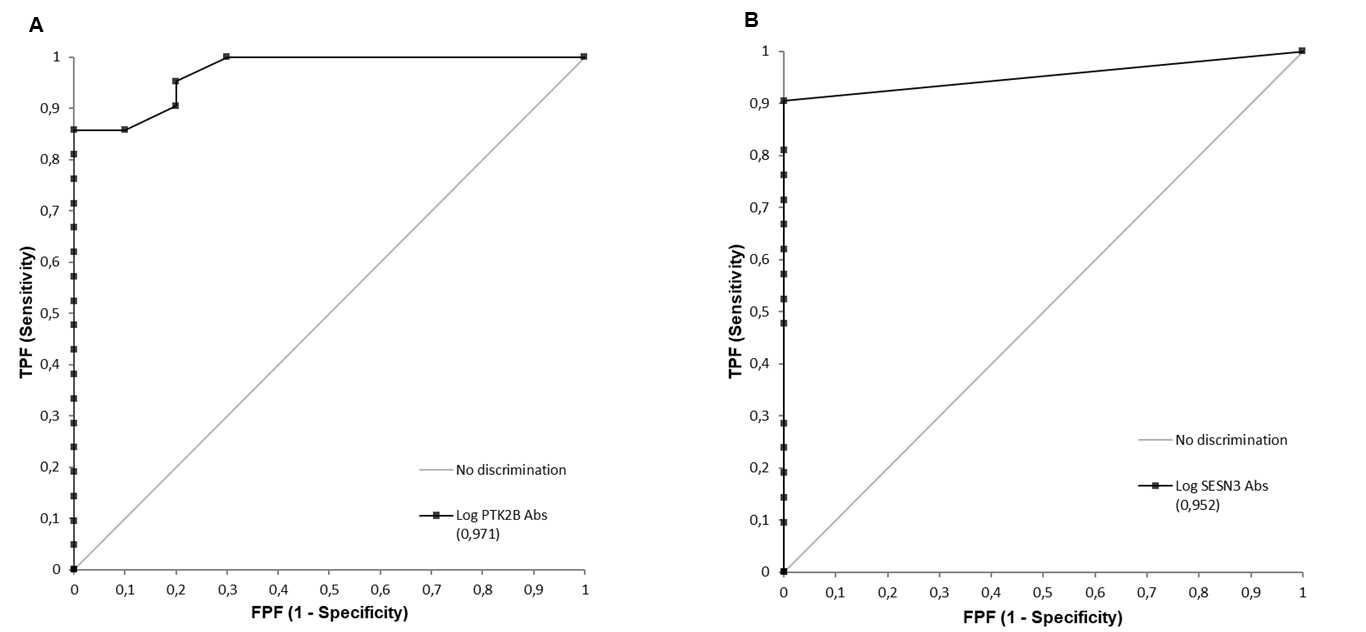

Supplement: Supplementary file 4 — Supplementary figure 3 [file 41409_2020_1090_MOESM4_ESM.tif]
